# Lessons and challenges from RA

**DOI:** 10.1186/1546-0096-12-S1-I21

**Published:** 2014-09-17

**Authors:** Philip Conaghan

**Affiliations:** 1 Leeds Institute of Rheumatic and Musculoskeletal Medicine, University of Leeds, Leeds, UK

## 

The application of modern imaging modalities such as magnetic resonance imaging (MRI) and ultrasonography (US) to rheumatoid arthritis (RA) has led to important advances in understanding of disease phenotype that are now impacting on diagnosis, monitoring and even therapy delivery. This presentation overviews the concepts derived from modern RA imaging, to underpin discussions of their relevance to juvenile idiopathic arthritis. Obviously there are particular issues for the application of MRI in children.

Modern imaging first demonstrated increased sensitivity for detection of both activity and damage measures above standard clinical examination and conventional radiography (CR). Both MRI and US have increased sensitivity for synovitis detection and in particular can distinguish synovitis and tenosynovitis. For erosions, both modalities have increased sensitivity above CR. MRI has better sensitivity in anatomical sites where US has a poor acoustic window, such as in the mid-carpus. The pathologies identified by both modalities have been validated against histology and other relevant techniques including CT. Uniquely MRI has demonstrated bone marrow oedema lesions, characterised histologically as osteitis. This lesion is highly predictive of subsequent erosion development. The increased sensitivity of MRI and US have provided evidence for a direct relationship between inflammation, its persistence and subsequent damage.

The next important message from modern imaging relates to what has been termed sub-clinical (perhaps better termed ‘non-clinically detected’) synovitis. A study in patients with low disease activity on clinician evaluation were studied with MRI and US of a single hand. About half the patients were in clinical remission criteria, but over 80% had synovitis in the studied hand on sensitive imaging. Follow up demonstrated this synovitis was important, in that it did result in erosion progression. Another study using US in oligoarticular arthritis found a large percentage of patients were re-classified as polyarticular when US criteria were employed. This has obvious implications for diagnosis and for pediatric disease.

Validity and reliability for assessment of pathologies with these modalities is now well evidenced. The sensitivity of these tools means they are well placed to objectively monitor therapeutic response. There has been a growing trial literature over the decade using both modalities, starting with smaller proof of concept studies that demonstrated that MRI and US could both demonstrate responses in keeping with large clinical trials, but in very small patient cohorts. There have been many advances in developing quantification for clinical trials, with responsiveness of the tools now established. Clearly there are strengths and weaknesses for each modality: for example MRI can give excellent synovitis and bone information, while US can evaluate more joints and is more patient-friendly. Challenges remain, especially about the optimal number of joints to assess.

The use of these tools in clinical practice has been restricted by access and training issues, with US being widely utilised now by adult rheumatologists across Europe, based on its immediacy in clinic, patient tolerance, ability to scan multiple joints and ability to guide intra-articular therapy. However cost-effective clinical algorithms are required for widespread use.

## Disclosure of interest

P Conaghan Consultant for: Abbvie, BMS, Janssen, Pfizer, Novartis, Roche, Speakers Bureau of: Abbvie, BMS, Janssen, Merck, Roche

